# A Comprehensive Review of Naringenin, a Promising Phytochemical with Therapeutic Potential

**DOI:** 10.4014/jmb.2410.10006

**Published:** 2024-11-22

**Authors:** Jun Hong Shin, Seung Ho Shin

**Affiliations:** 1 Department of Food and Nutrition, Gyeongsang National University, Jinju 52828, Republic of Korea; 2 Department of Bio & Medical Bigdata (BK4 Program), Gyeongsang National University, Jinju 52828, Republic of Korea

**Keywords:** Phytochemical, naringenin, cancer, metabolic disorder, neurodegenerative disease

## Abstract

Disorders, including cancer, metabolic disorders, and neurodegenerative diseases, can threaten human health; therefore, disease prevention is essential. Naringenin, a phytochemical with low toxicity, has been used in various disease prevention studies. This study aimed to comprehensively review the effects of naringenin on human health. First, we introduced the general characteristics of naringenin and its pharmacokinetic features when absorbed in the body. Next, we summarized the inhibitory effects of naringenin on colorectal, gastric, lung, breast, ovarian, cervical, prostate, bladder, liver, pancreatic, and skin cancers in preclinical studies. Lastly, we investigated the inhibitory effects of naringenin on metabolic disorders, including diabetes, obesity, hyperlipidemia, hypertension, cardiac toxicity, hypertrophy, steatosis, liver disease, and arteriosclerosis, as well as on neurodegenerative diseases, including Alzheimer's disease and Parkinson's disease. In conclusion, naringenin may serve as a significant natural compound that benefits human health.

## Introduction

Advances in medical technology have increased human life expectancy; however, aging and westernized eating habits have led to the development of various diseases, including cancer, metabolic disorders, and neurodegenerative diseases. Cancer is one of the leading causes of death in humans, and its prevalence is expected to continue to increase, according to the World Health Organization (WHO) [1]. Cancer is treated by a combination of treatments, including surgery and chemotherapy; however, owing to the side effects, prevention through healthy eating is more essential [2, 3]. Furthermore, healthy eating habits are crucial in alleviating metabolic disorders, including obesity. Metabolic disorders can significantly lead to cardiovascular diseases and subsequent death [4]. Increased reactive oxygen species (ROS) due to aging can induce neurodegenerative diseases, including Alzheimer's disease (AD) and Parkinson's disease (PD) [5-7]. Prevention of neurodegenerative diseases is essential because the cure is impossible, and the cause remains unclear [8].

Polyphenols, which can be used as a solution for suppressing the onset of diseases, are naturally derived compounds with low toxicity and beneficial effects [9-11]. Polyphenols are abundant in vegetables and fruits and have antioxidant activity, showing beneficial effects in various diseases [12, 13]. Polyphenols include flavonoids and non-flavonoids [14]. Naringenin, a member of the flavonoid family, is a flavanone mainly noted in citrus fruits [15]. Naringenin has antioxidant, anti-inflammatory, and anti-viral effects and lowers the risk of cardiovascular disease, metabolic syndrome, and cancer [16].

We here comprehensively review the various effects of naringenin. First, we describe the sources of naringenin and its characteristics. Second, we discuss the pharmacokinetic aspects of naringenin and explain how it is absorbed, distributed, metabolized, and extracted in the body. Third, we discuss the effects of naringenin on cancer, metabolic disorders, and neurodegenerative diseases. Additionally, we summarize the inhibitory effects of naringenin on colorectal, gastric, lung, breast, ovarian, cervical, prostate, bladder, liver, pancreatic, and skin cancers. Diabetes, obesity, hyperlipidemia, hypertension, cardiac toxicity, hypertrophy, steatosis, liver disease, and arteriosclerosis are described in the metabolic disorder section. Finally, we here discuss the effects of naringenin on neurodegenerative diseases, including AD and PD.

## Natural Sources of Naringenin

**Fig. 1:**
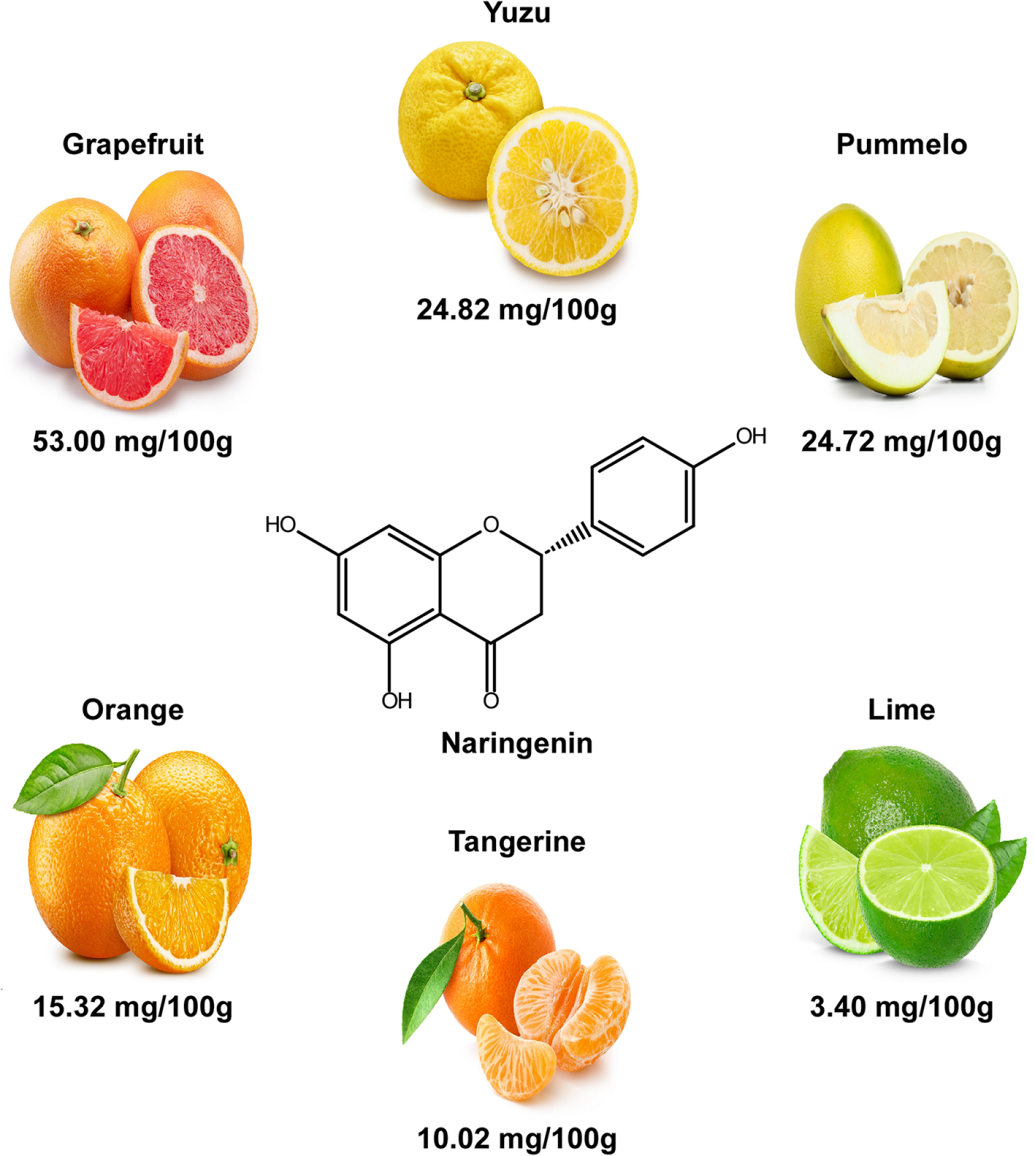
The structure and natural sources of naringenin.

Naringenin ((2S)-5,7-dihydroxy-2-(4-hydroxyphenyl)-2,3-dihydrochromen-4-one) is a flavanone, a type of flavonoid, and is colorless and odorless (Fig. 1) [17, 18]. With a molecular weight of 272.256 g/mol and a melting point of 251°C, naringenin demonstrates favorable solubility in organic solvents, including ethanol, dimethylformamide, and dimethyl sulfoxide. Conversely, its solubility in buffered aqueous solutions is limited, reaching approximately 475 mg/l [19-21].

Naringenin is the most abundant in grapefruit, with a content of 53.00 mg/100 g. The contents of naringenin in other fruits are as follows: yuzu, 24.82mg/100g; pummelo, 24.72mg/100 g; orange, 15.32 mg/100 g; tangerine, 10.02 mg/100 g; and lime, 3.40 mg/100 g (Fig. 1) [22].

Moreover, in humans, the naringenin content is crucial owing to its conversion into naringenin by naringinase. This breakdown process occurs in two steps. First, naringinase exhibits α-L-rhamnosidase activity, thereby leading to naringin hydrolysis into rhamnose and prunin. Second, prunin is further hydrolyzed by the β-D-glucosidase activity of naringinase, subsequently forming naringenin and glucose [23].

Citrus fruits, such as musk lime, Mexican lime, rough lime, pummelo, and mandarin orange, are abundant in naringin, similar to naringenin. Pummelós peel (3,910 μg/g) contains more naringin content than its juice (220 μg/g). Similarly, the peel, juice, and seeds of rough lime have naringin contents of 517, 98, and 29 μg/g, respectively [24].

## Pharmacokinetics of Naringenin

### Absorption

Naringenin is absorbed in the duodenum, jejunum, ileum, cecum, and colon; however, its systemic absorption rate is limited [25, 26].

Studies conducted in human intestinal Caco-2 cells [27] have reported that naringenin is partially absorbed through passive diffusion, and pH changes do not affect its absorption. Furthermore, it has been identified as a substrate for adenosine triphosphate (ATP)-dependent transport facilitated by multidrug resistance-associated protein 1. Another study using a murine intestinal tract model [28] has shown that the highest absorption rate (68%) of naringenin occurred in the colon. The following were the absorption rates in different parts of the intestine: duodenum 47%; terminal ileum 42%; and jejunum 39%. Moreover, a pharmacokinetic study in humans [29] has noted the following parameters related to a 135-mg naringenin oral dose: area under the plasma concentration-time curve (AUC_0-∞_) of 9,424.52 ng h/mL; elimination half-life, 2.31 h; and relative cumulative urinary excretion, 5.81%.

### Distribution

Naringenin can be distributed to various organs, including the brain, liver, kidney, spleen, and heart [30].

β-Glucuronidase–enriched sulfatase primarily hydrolyzes naringenin to glucuronide and sulfate forms [31]. The glucuronide form is predominantly present in the serum. However, in tissues including the brain, heart, liver, and pancreatic tissues, it is present in a sulfated form, indicating glucuronidation and subsequent sulfation within these organs [31].

Various studies have shown that different flavonoids can cross different brain regions to varying extents. One study utilized an established ECV304 cell model for *in vitro* permeability assessment, cocultured with C6 glioma cells to mimic the *in vivo* glial inductive effect on the blood-brain barrier (BBB). The ECV304 cells exhibited brain endothelial features, including elevated transendothelial electrical resistance, restricted junctional complexity, and glucose transporter (GLUT)-1 expression. Interestingly, the study indicated that pretreatment with various efflux transporters did not influence naringenin [32, 33].

Peng *et al*. investigated naringenin uptake and transport in the cerebral cortex and striatum and observed that the striatum had reduced levels compared with the cerebral cortex. In the *in vitro* model, the apparent permeability of naringenin was between 250 and 350 nm/s, indicating its high permeability across both *in situ* BBB models and *in vitro* studies [34].

### Metabolism

To form aglycones, including apigenin, apiferol, eriodictyol, and hesperetin, naringenin undergoes dehydrogenation, hydrogenation, hydroxylation, and methylation. Naringenin and its aglycones are sulfated or glucuronated by phase II metabolic enzymes in the stomach, liver, and other tissues. Thirty-nine flavonoid metabolites are generated by naringenin and its derivatives, including apigenin, apiferol, eriodictyol, and hesperetin, through sulfation or glucuronidation. These metabolites exist as *O*-glucuronide, *O*-diglucuronide, *O*-sulfate, *O*-disulfate, *O*-glucuronide-sulfate, *O*-glucoside-*O*-glucuronide, and *O*-glucoside-*O*-sulfate [35].

Moreover, unabsorbed flavonoids produce phenolic catabolites within the gut microbiome. Forty-six phenolic catabolites were identified, including phenylpropenoic acid, phenylpropionic acid, phenylacetic acid, benzoic acid, benzenetriol, and benzoylglycine derivatives [35].

Under anaerobic conditions, when cultured with human fecal solutions, naringenin undergoes metabolism for >24 h to yield HPPA, 3-(phenyl)propionic acid, and minor quantities of 3-(4'-hydroxyphenyl)acetic acid [36]. The NADH-dependent reductase enzyme of the human colonic anaerobe *Eubacterium ramulus* is responsible for cleaving the heterocyclic C-ring of naringenin [37].

The degradation of flavanones, including hesperetin-7-*O*-rutinoside, naringenin-7-*O*-rutinoside, hesperetin, and naringenin, was investigated by coculturing with probiotic bacteria *Bifidobacterium longum* (*B. longum*) and *Lactobacillus rhamnosus* in orange juice [38]. These bacteria induce the ring cleavage, demethylation, or dehydrogenation of flavanones, forming 3-(phenyl)propionic acid. Long-term *B. longum* R0175 administration of >4 weeks increased urinary excretion of metabolites and organic acids derived from orange juice flavanones, indicating enhanced bioavailability [39].

### Excretion

Naringenin is excreted via the following two routes: urine and bile. Initially, approximately 1%–30% of the ingested naringenin is excreted in the urine. Differences in urinary excretion may be due to individual differences in liver function and differences in intake according to the naringenin level, which is more abundant in citrus peels [40].

Naringenin glucuronides, especially M2, are observed in bile, whereas naringenin sulfate is not detected. Moreover, the hepatic metabolism of naringenin glucuronide is more efficient than the intestinal metabolism [41]. Naringenin glucuronides are predominantly absorbed in the upper small intestine, with approximately 27% and 18% excreted in the duodenum and jejunum. Moreover, efflux transporters MRP2 and breast cancer resistance protein-1 compensate for each other, enabling the intraintestinal excretion of flavonoid glucuronides, including naringenin [41].

## Preclinical Studies of Naringenin

### Cancer

Cancer is a significant cause of mortality worldwide, with its incidence anticipated to increase globally, particularly in low and middle-income countries [42]. Incorporating vegetables and fruits into the diet has been suggested as a promising strategy for preventing cancer. A study encompassing 34 varieties of citrus juices examined their effects on the cell lines of the following four cancer types: lung carcinoma, melanoma, leukemia, and gastric carcinoma [43]. When citrus fruit flavonoids were administered to the same cell lines, naringin and naringenin demonstrated antiproliferative effects starting from a 0.04 mM concentration. Notably, naringenin exhibited more vital growth inhibitory properties than naringin [44]. Here, we addressed colorectal, gastric, lung, breast, ovarian, cervical, prostate, bladder, liver, pancreatic, and skin cancers. We reviewed the *in vivo* studies and summarized the significant mechanisms elucidated *in vitro*.

**Fig. 2:**
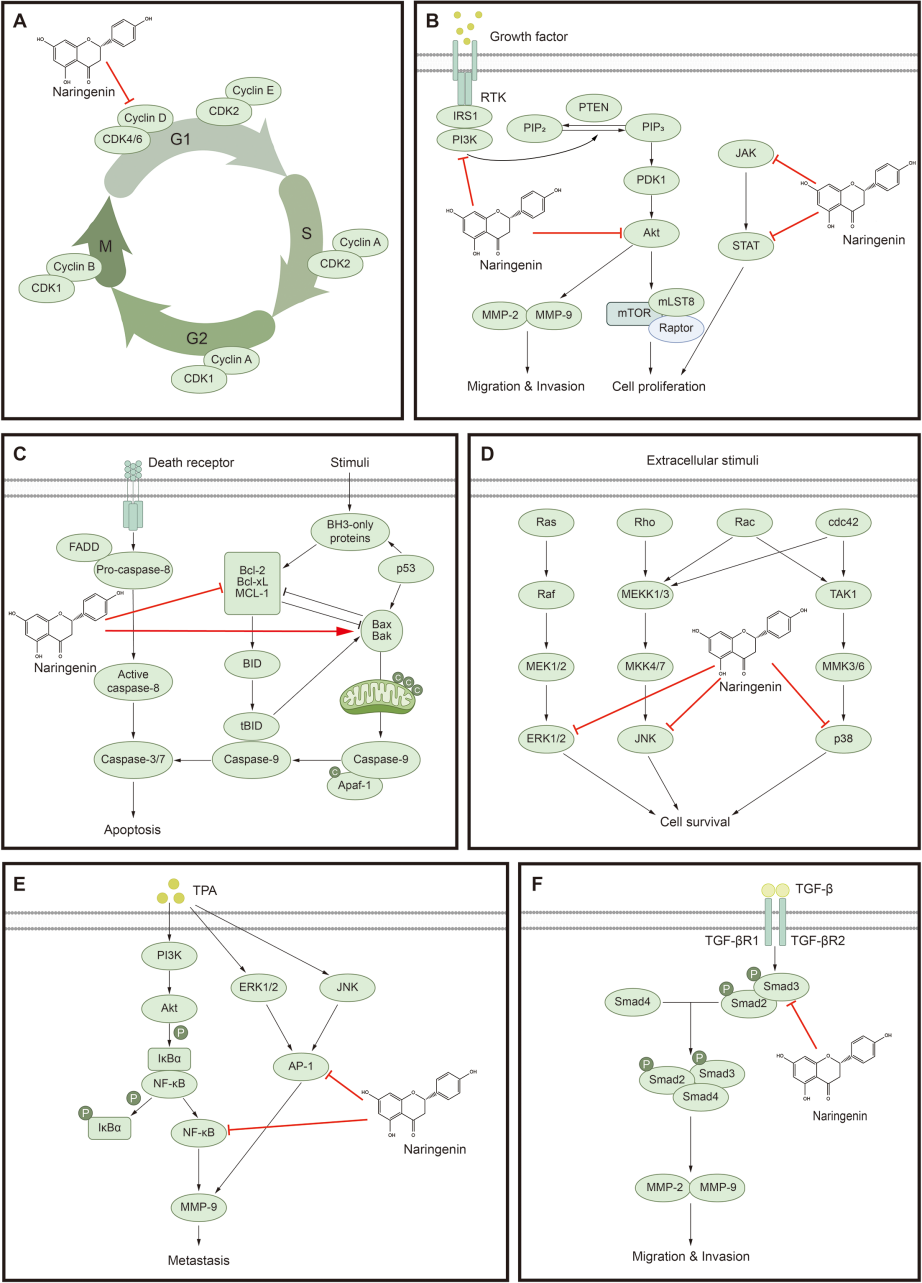
The signaling pathways of naringenin in cancer. (**A**) Naringenin arrests the cell cycle by inhibiting cyclin D. (**B**) Naringenin inhibits cell proliferation by inhibiting the JAK/STAT pathway and suppresses cell proliferation, migration and invasion by inhibiting the PI3K/Akt pathway. (**C**) Naringenin induces apoptosis by regulating pro- and anti-apoptosis factors. (**D**) Naringenin decreases cell survival by inhibiting ERK1/2, JNK, and p38. (**E**) Naringenin attenuates metastasis by inhibiting the activity of NF-κB and AP-1. (**F**) Naringenin decreases cell migration and invasion by inhibiting the activity of Smad-3.

**Colorectal and gastric cancers.** Colorectal cancer occurs in the colon and rectum, ranking as the third leading cause of cancer-related deaths in the United States in 2023 [45]. As dietary habits play an essential role in the pathogenesis of colorectal cancer, dietary chemotherapy has attracted attention for colorectal cancer prevention [46]. A previous study has demonstrated the protective effects of citrus flavonoids against colorectal cancer and reported that naringenin inhibited HT-29 colon cancer cell proliferation [47]. Treatment with 6-C-(E-phenylethenyl)naringenin (6-CEPN) reduced the levels of autophagy-related protein 7 and beclin-1, which are crucial proteins involved in autophagy in colorectal cancer, thereby inducing autophagy and apoptosis by arresting cell proliferation at the G1 phase of the cell cycle [48]. Treatment with naringenin reduced cyclin D1 levels in the HCT116 and SW480 colorectal cancer cell lines (Fig. 2A) [49]. Moreover, recent studies have demonstrated that loading naringenin into nanostructured lipid carriers with a 98-nm particle size enhances its bioavailability and cellular absorption, serving as a potent trigger for cell apoptosis in HT-29 cells [50].

In 2020, the fifth most diagnosed cancer and the fourth leading cause of cancer-related death worldwide was gastric cancer [45, 51]. The causes of gastric cancer include *Helicobacter pylori* infection, genetic factors, alcohol consumption, and smoking [52-55]. Naringenin inhibited cell proliferation, migration, and invasion by downregulating Akt in SGC7901 gastric cancer cells [56]. Naringenin inhibited SGC7901 cell proliferation and reduced the proliferating nuclear antigen level. Akt pathway downregulation was the primary mechanism, and naringenin inhibited the cell migration, invasion, and expression of matrix metalloproteinase (MMP)-2 and MMP-9 (Fig. 2B). The expression of BAX and cleaved caspase-3 increased, whereas that of Bcl-2 decreased (Fig. 2C). Additionally, the combined administration of naringenin and the Akt inhibitor, LY294002, showed an improved effect.

**Lung cancer.** Lung cancer is a commonly diagnosed cancer, accounting for approximately 11.6% of all cancer diagnoses [57]. In 2023, approximately 238,340 new cases of lung cancer would be diagnosed in the United States, and nearly 127,070 individuals would be die from the disease [45]. Lung cancer is the primary cause of cancer-related death (accounting for 18.4% of all cancer-related deaths), causing severe economic burden and social difficulties [45, 58]. Smoking is the primary cause of lung cancer, with asbestos exposure, air pollution, chronic polycyclic aromatic hydrocarbon exposure, and genetic predisposition as additional factors [59]. Naringenin oral administration significantly reduced the number of metastatic tumor cells in the lungs and extended the lifespan of tumor-resected mice. Moreover, naringenin increased the proportion of T cells expressing interferon-γ and interleukin-2 and enhanced antitumor activity [60]. In mice with pulmonary fibrosis, a 100 mg/kg naringenin dose decreased the risk of lung metastasis. Naringenin treatment increased the levels of transforming growth factor (TGF)-β1 and CD4^+^CD25^+^Foxp3^+^ regulatory T cells [61].

*In vitro* studies using the A587 lung cancer cell line with naringenin have demonstrated cell migration inhibition. This effect is attributed to the inhibition of MMP-2, MMP-9 and Akt activity, which are crucial for cancer cell migration [62]. Tumor necrosis factor-related apoptosis-inducing ligand (TRAIL) selectively triggers apoptosis in cancer cells with minimal harm to normal cells. However, some non-small-cell lung cancer (NSCLC) cells show resistance. When combined with TRAIL, naringenin induced apoptosis in TRAIL-resistant A549 NSCLC cells. The naringenin and TRAIL combination suggests a potentially safe therapy for NSCLC [63]. Furthermore, the NSCLC cell lines, A549 and H1299, showed synergistic antiproliferative effects of naringenin and apigenin [64]. A 2:3 mixture of naringenin and apigenin induced potent cytotoxicity and G2/M cell cycle arrest. Compared with either apigenin or naringenin treatment alone, the naringenin and apigenin combination therapy potentiated mitochondrial dysfunction, increased oxidative stress, and activated the apoptotic pathways [64].

**Breast cancer.** In 2020, breast cancer was the most common malignancy among females, accounting for 11.7%of new cancer cases worldwide [51]. In a breast cancer mouse model, naringenin enhanced antitumor activity when administered with doxorubicin and metformin compared with doxorubicin alone [65]. In a mouse model, the co-administration of naringenin with cryptotanshinone reduced JAK2/STAT3 phosphorylation and decreased the CD4^+^CD25^+^Foxp3^+^ T cell population within the tumor (Fig. 2B) [66]. Naringenin decreased TGF-β1 secretion levels in breast cancer cells and inhibited the metastasis of lung tumors [67]. Moreover, the inhibition of 4T1 tumor metastasis increased survival in mice. Naringenin did not affect TGF-β1 transcription; however, it hindered its transport from the trans-Golgi network [67].

In *in vitro* studies, naringenin reduced MDA-MB-231 breast cancer cell viability, which significantly increased caspase-3 and caspase-9 activity, thereby promoting cell apoptosis [68]. Another study has evaluated the effects of naringenin on insulin-induced glucose uptake in proliferation and growth-arrested MCF-7 breast cancer cells [69]. Results revealed that naringenin suppressed the activity of PI3K, insulin-induced GLUT-4 translocation, and p44/p42 MAPK phosphorylation, leading to a 60% reduction in insulin-stimulated glucose absorption, thereby inhibiting MCF-7 cell proliferation [69]. Additionally, naringenin inhibited the growth potential of MDA-MB-231 breast cancer cells and downregulated the MMP-2 and MMP-9 expression by binding to Integrin β3, thereby blocking breast cancer cell movement and invasion [70].

**Ovarian and cervical cancers.** In 2020, ovarian cancer accounted for 1.6% of new cancer diagnoses and 2.1% of all cancer-related deaths worldwide [51]. It mainly developed in postmenopausal females and was primarily caused by mutations in the *BRCA1* and *BRCA2* genes [71]. In *in vitro* studies, naringenin treatment effectively inhibited A2780 and ES-2 ovarian cancer cell line proliferation and migration by downregulating PI3K [72]. In *in vivo* studies, naringenin administration significantly reduced tumor weight and volume, with oral administration showing superior efficacy compared with intraperitoneal injection. Furthermore, the microbial composition was improved by naringenin therapy, markedly increasing the abundance of *Alistipes* and *Lactobacillus* [72].

In 2020, cervical cancer accounted for 3.1% of all cancer diagnoses and comprised 3.4% of all cancer-related deaths worldwide [51]. Infection with oncogenic subtypes of the human papillomavirus was the primary causative factor [73]. Owing to the low naringenin bioavailability, studies were conducted in combination with nanoparticles in human cervical cancer HeLa cells [74]. Naringenin-loaded nanoparticles (NARNPs) exhibited more significant cell toxicity than naringenin alone. NARNPs increased intracellular ROS levels and lipid peroxidation status while reducing glutathione (GSH) levels. Furthermore, NARNPs treatment led to MMP alterations and an increased apoptotic index in cancer cells. These results underscore the potential of NARNPs as a promising strategy for potential anticancer therapy in cervical cancer [74].

**Bladder and prostate cancer.** In 2020 bladder cancer was the 10th most common cancer worldwide, with an annual incidence of 573,000 cases and 212,536 deaths [51]. It occurs more frequently in males than females, and the incidence increases with age [75]. Naringenin treatment for 24 h reduced cell viability in TSGH8301 bladder cancer cells [76]. Furthermore, by downregulating MMP-2 and Akt activities, naringenin dose-dependently decreased TSGH8301 cell migration [76].

Prostate cancer is the most common type of male urogenital malignancy, and risk factors, including genetic predisposition, race, and age, are previously reported [45, 77]. Naringenin can reverse the expression of proteins involved in the epithelial-mesenchymal transition (EMT) in human prostate cancer cells, specifically PC-3 cells, and inhibit the activity of urokinase plasminogen activator, thereby leading to cell migration suppression [78]. Moreover, naringenin treatment inhibited cell proliferation and reduced cell motility in MAT-LyLu prostate cancer cells. Naringenin inhibited cell migration by reducing *SCN9A* gene expression [79]. Additionally, naringenin increased MMP and BAX loss while decreasing Bcl-2 protein levels in PC-3 cells [80]. In LNCaP cells, naringenin decreased ERK1/2, P53, P38, and JNK protein phosphorylation in PC3 cells, it decreased ERK1/2, P70S6K, S6, and P38 phosphorylation (Fig. 2D). This finding indicates the potential anticancer effects of naringenin through the PI3K/Akt and MAPK signaling pathways [80].

**Liver cancer.** Hepatocellular carcinoma (HCC) associated with fibrosis and chronic inflammation is influenced by various risk factors, including alcohol consumption, aflatoxin B1, hepatitis B/C virus, infection, and metabolic disorders [81]. In a rat model of liver cancer induced by N-nitroso diethylamine (NDEA), the efficacy of naringenin was evaluated [82]. Following NDEA-induced HCC, naringenin pre- and post-treatment modulated xenobiotic metabolism enzymes, attenuated lipid peroxidation, and reduced liver marker enzyme levels [82].

Naringenin showed potent anticancer effects in diethylnitrosamine-induced HCC cell lines [83]. Additionally, naringenin inhibited the 12-*O*-tetradecanoylphorbol-13-acetate (TPA)–induced invasion in human liver cancer cell lines (HepG2, Huh-7, and HA22T) and rat embryonic liver cells (BNLCL2) [84]. Naringenin inhibited MMP-9 secretion; this inhibition was mediated by reducing MMP-9 transcription by inhibiting activator protein (AP)-1 and NF-κB activities (Fig. 2E). Naringenin inhibited the phosphorylation of the ERK and JNK signaling pathways. Moreover, it inhibited TPA-induced ERK/PI3K/Akt activation upstream of NF-κB and AP-1. These findings suggest that by targeting multiple signaling pathways, naringenin can inhibit the invasiveness and metastatic potential of HCC [84]. 6-CEPN, a semisynthetic derivative of naringenin, reduced cell viability and inhibited sphere formation, cell migration, and invasion of HCC cell lines. Furthermore, it inhibited the EMT of HCC stem cells and simultaneously inhibited the Wnt/β-catenin signaling pathway [85].

**Pancreatic cancer.** Pancreatic cancer is a highly dire and aggressive tumor, being one of the most perilous cancers with a survival rate of only 7% [86, 87]. By suppressing the TGF-β signaling pathway, a central regulator of EMT, naringenin inhibited pancreatic cancer. In addition, it suppressed migration through caspase-3 cleavage, increased ROS levels, and induced cell death via apoptosis signal-regulating kinase (ASK)-1 mediation. First, by inhibiting the TGF-β/Smad-3 signaling pathway, naringenin reduced EMT marker levels (Fig. 2F) [87]. TGF-β is a pivotal regulator of EMT, governing cellular motility, transition, and invasion, and Smad-3 regulates it. Moreover, naringenin augmented the sensitivity of PANC-1 pancreatic cancer cells to gemcitabine, the most potent drug used in pancreatic cancer clinical therapy [87]. Second, the combination treatment of naringenin and hesperetin suppressed migration in PANC-1 pancreatic cells and inhibited FAK and p38 phosphorylation [88]. This study was conducted by treating PANC-1 pancreatic cells with a naringenin and hesperetin combination. The combination of these two compounds targeted caspase-3 cleavage, thereby inhibiting PANC-1 pancreatic cell migration and suppressing FAK and p38 phosphorylation, which was not observed with individual treatments [88]. Lastly, naringenin increased ROS levels in SNU-213 pancreatic cancer cells, thereby triggering ASK-1-mediated cell death [89]. Treating SNU-213 cells with naringenin reduced the expression of p38, JNK, p58, and peroxiredoxin-1, an oxidative stress cell homeostasis regulator.

**Skin cancer.** Skin cancer, which has several types, is the most commonly diagnosed cancer in the United States. The most common types of skin cancer are non-melanoma skin cancer, basal cell carcinoma, and squamous cell carcinoma; however, they rarely cause death or severe morbidity. Melanoma accounts for approximately 1% of all skin cancers but is the leading cause of skin cancer deaths [90]. In skin cancer, naringenin inhibits glyoxalase-1 activity, increases ROS production, induces apoptosis, and inhibits melanoma metastasis by inhibiting two-pore channel 2 (TPC2). First, naringenin induced apoptosis in A431 human skin cancer cells [91]. In addition, it increased ROS production, induced cell cycle arrest in the G0/G1 phase, and enhanced caspase-3 activity. Second, in a skin papilloma mouse model, the preventive effects of naringenin were evaluated [92]. In both pre- and post-treatment models, naringenin reduced the skin papilloma. Biochemical studies have reported that naringenin decreased glyoxalase-1 activity, indicating that it increases oxidative damage in tumors. Lastly, naringenin inhibited TPC2 and melanoma cell angiogenesis [93]. Naringenin inhibited TPC2 and VEGF angiogenesis activation by interfering with intracellular calcium signaling.

### Metabolic Disorders

Metabolism is the highly regulated process of separating consumed food into simple components, including carbohydrates, proteins, and fats [94]. Diabetes, obesity, hyperlipidemia, hypertension, cardiac toxicity, hypertrophy, hyperglycemia, steatosis, hepatic protection, and atherosclerosis are the most common metabolic disorder-related diseases. This chapter will introduce the efficacy of naringenin in treating metabolic disorders.

**Diabetes.** Diabetes is a severe, non-infectious endocrine metabolic disorder that can lead to complications in multiple organs [95]. Diabetes is characterized by elevated blood glucose levels due to insufficient insulin secretion by pancreatic β cells or increased insulin resistance to glucose [96, 97]. Diabetes can lead to several complications, including renal failure, liver dysfunction, blindness, cardiac arrest, stroke, and neurological damage [94, 98, 99]. Therefore, maintaining normal blood glucose levels as a preventive measure against diabetes is imperative.

In *in vivo* models, naringenin reduced blood sugar levels and increased insulin sensitivity. Naringenin reduced blood glucose, total cholesterol, and triglyceride (TG) levels in a streptozotocin (STZ)-induced Wistar rat model. Treatment with naringenin 50 mg/kg increased high-density lipoprotein (HDL) cholesterol levels [100]. In another STZ-induced mouse model, naringenin decreased the levels of blood glucose and various metabolic parameters while increasing serum insulin levels [101]. Naringenin lowered cholesterol levels and improved hematological and immune parameters, including red blood cells, hemoglobin, ALKP, urea, and TG, in alloxan-induced diabetic rats [102]. Furthermore, naringenin improved insulin sensitivity and enhanced tyrosine phosphorylation in Wistar rats with type 2 diabetes (T2D) induced by a high-fructose diet. Naringenin enhanced tyrosine phosphorylation, which allows naringenin to be considered an effective insulin sensitizer [103]. In albino Wistar rats with T2D induced by a high-fat diet, naringenin delayed carbohydrate absorption by inhibiting α-glucosidase activity [104]. Moreover, naringenin significantly reduced postprandial blood sugar levels [104]. In a high-glycemic diet-induced rodent model, naringenin increased GLUT-4 translocation [105]. Additionally, naringenin increased AMP-activated protein kinase (AMPK) phosphorylation and SIRT1 and PGC-1α expression [105]. Furthermore, in genetic animal models, naringenin demonstrated antidiabetic effects. An Ldlr^−/−^ mouse model is prone to obesity and hyperlipidemia, which leads to T2D. In the Ldlr^−/−^ diabetic mouse model, naringenin treatment reduced fasting plasma glucose, insulin, and cholesterol levels [106].

In *in vitro* studies, naringenin increased glucose uptake, and GLUT-4 translocation in muscle cells increased insulin secretion and improved the survival of pancreatic beta cells. In L6 muscle cells, naringenin increased glucose uptake [107]. Additionally, naringenin enhanced AMPK phosphorylation, and AMPK siRNA attenuated the effects of naringenin [107]. In another study on palmitate-induced insulin-resistant L6 myotubes, naringenin increased insulin-stimulated glucose uptake and GLUT-4 translocation [105]. Moreover, naringenin increased SIRT1 and PGC-1α levels [105]. In rat pancreatic INS-1E cells, naringenin increased glucose-stimulated insulin secretion and β-cell gene expression [108].

**Obesity and hyperlipidemia.** Overweight and obesity are characterized by abnormal or excessive fat accumulation that can pose health risks. A body mass index >25 kg/m^2^ is considered overweight, and >30 kg/m^2^ is considered obese. In the United States, 40% of the population is considered obese [109, 110]. Hyperlipidemia is characterized by abnormally high levels of lipids and cholesterol in the blood, predisposing the individual to atherosclerosis and other arterial diseases. Hyperlipidemia is diagnosed when the total cholesterol, low-density lipoprotein (LDL)-cholesterol, and TG levels are ≥240, ≥160 mg/dL, ≥150 mg/dL, respectively [111]. Obesity is a major cause of metabolic syndrome, including diabetes, high blood pressure, and hyperlipidemia, ultimately causing atherosclerosis and cardiovascular diseases [112, 113]. The prevalence of obesity worldwide is mainly due to a high-fat-, high-sugar-, westernized diet, highlighting the significance of obesity prevention [114].

In *in vivo* studies, obesity and hyperlipidemia were effectively improved by naringenin treatment. After consuming a diet containing naringenin for 6 months, compared with the control group, serum and liver cholesterol levels, as well as the content of neutral sterols in stool, were reduced [115]. Additionally, lipid levels were lowered by reducing 3-hydroxy-3-methylglutaryl coenzyme A and acyl-coenzyme A: cholesterol *O*-acyltransferase activities [115]. Normal cholesterol levels were maintained by adding naringenin to a high-fat diet [116]. Moreover, by upregulating the expression of peroxisome proliferator-activated receptor (PPAR) α and its downstream factors, carnitine–palmitoyl transferase 1L and uncoupling protein 2, in the liver, naringenin showed an anti-steatosis effect [116]. In high-fat diet-induced obese mice, treatment with naringenin 10 mg/kg upregulated the genes associated with lipolysis, synthesis, and serum and hepatic lipid metabolism [117]. In C57BL/6 mice with high-fat diet-induced obesity, naringenin suppressed the expression of toll-like receptor 2, which is associated with obesity-induced inflammation that causes insulin resistance and T2D [118]. In a high-fat diet-induced obesity SD rat model, the treatment of naringenin 100, 200, and 400 mg/kg reduced the total cholesterol, TG, and non-HDL cholesterol levels [119]. Furthermore, in SD rats, naringenin alleviated hepatic steatosis and reduced the epididymal adipocyte size [119]. In Ldr^^−/−^^ mice, 3% naringenin also improved cholesterol-induced hyperlipidemia, inflammation, and obesity-induced atherosclerosis [120]. Naringenin supplementation to both Ldr^^−/−^^ and C57BL/6J mice fed a high-fat diet suppressed insulin resistance, lipid metabolism abnormalities, and obesity compared with controls [121].

In *in vitro* studies, naringenin reduced TG and cholesterol levels and increased adipocyte protein expression. In 3T3-L1 adipocytes, naringenin reduced total cholesterol and TG levels and increased AMPK phosphorylation [119]. In 3T3-L1 adipocytes, naringenin reduced lipid accumulation and upregulated the expression of adipocyte proteins, including STAT5A and PPAR [122].

**Hypertension.** Hypertension is a chronic disease characterized by increased blood pressure and is a significant cause of cardiovascular diseases. Hypertension affects at least 1.4 billion individuals worldwide [123, 124]. Hypertension is a significant risk factor for coronary heart disease, stroke, and chronic kidney disease and leads to premature mortality and morbidity [125]. Therefore, preventing high blood pressure is highly significant.

In *in vivo* studies, naringenin exhibited antihypertensive effects by lowering blood pressure and inhibiting the JAK2/STAT3 pathway and angiotensin (Ang)-converting enzyme (ACE). In a rat model of high-fat diet-induced hypertension, 50 and 100 mg/kg of naringenin treatment reduced blood pressure and modulated serum lipid parameters by decreasing cholesterol, TG, and LDL levels and increasing HDL levels [126]. Additionally, naringenin decreased serum malondialdehyde (MDA) and nitrite oxide levels, increased superoxide dismutase (SOD) and GSH levels, regulated adipocytokine levels, and decreased STAT3 phosphorylation [126]. In a mouse model of pregnancy-induced hypertension, naringenin reduced the blood pressure, and the levels of total urine protein, vasodilation-converting enzyme, α-1A adrenergic receptor, and Ang [127]. In mouse vascular endothelial cells, naringenin suppressed the JAK2/STAT3 signaling pathway by inhibiting the expression of the Src homology 2 domain-containing protein tyrosine phosphatase 1 [127]. In an animal model of renovascular hypertension through two-kidney one-clip surgery, naringenin 200 mg/kg treatment delayed the elevation of Ang 2 levels in the peripheral blood [128]. Additionally, the increase in the ACE/ACE2 protein ratio and the Ang 1 receptor/Ang 2 receptor protein ratio was inhibited by naringenin [128]. In the L-NG-Nitro arginine methyl ester-induced rat model, naringenin treatment exhibited antihypertensive and neuroprotective effects through the downregulation of renal injury molecule 1, mineralocorticoid receptor, and ACE [129].

**Hyperglycemia.** Hyperglycemia is characterized by a fasting blood sugar level of >125 mg/dL and a 2-h postprandial blood sugar level of >180 mg/dL [130-132]. Decreased insulin secretion, reduced glucose utilization, and increased glucose production are the factors contributing to hyperglycemia [132, 133]. The latest data released by the Centers for Disease Control and Prevention revealed that approximately 30.5 and 84 million Americans have diabetes and pre-diabetes, respectively [134].

Naringenin alleviates hyperglycemia by reducing blood sugar levels and protects against inflammation and oxidative stress caused by hyperglycemia. In a Wistar rat model of STZ and nicotinamide-induced diabetes, the hyperglycemia-induced inflammation was alleviated by naringenin [135]. Daily IP treatment with naringenin 50 mg/kg improved hematological indicators, including erythrocyte sedimentation rate, total white blood cell (WBC) count, differential WBC percentage, and platelet count [135]. Moreover, naringenin decreased the level of the pro-inflammatory cytokine, NF-κB [135]. Compared with diabetic rats that did not receive naringenin, those that received naringenin treatment showed decreased fasting blood sugar and glycated hemoglobin levels and increased serum insulin levels [136]. Hyperglycemia was induced by treating Chang cells with glucose, and naringenin treatment increased cell survival and reduced oxidative stress [137]. In an STZ-induced diabetes Sprague-Dawley rat model, naringenin treatment reduced the levels of nuclear factor erythroid 2-related factor 2 (Nrf2) and oxidative stress [137]. When STZ-induced diabetic rats were fed a high-fat diet and subsequently treated with naringenin, hyperglycemia and hyperlipidemia were improved [138]. In addition, treatment with naringenin increased GLUT-4 expression and decreased TNF-α expression [138].

**Steatosis and liver disease.** Steatosis is characterized by fat accumulation in organs, such as the liver. The normal liver also contains fat but becomes impaired when the fat content exceeds 5% [139]. Nonalcoholic fatty liver disease (NAFLD) is divided into the early stage, nonalcoholic fatty liver (NAFL), and the worsening stage, nonalcoholic steatohepatitis [140]. NAFLD is related to metabolic syndrome, and more than one-third of patients with type 2 diabetes mellitus develop NAFLD [141, 142].

Naringenin inhibits fat accumulation in the liver, thereby alleviating liver diseases, including steatosis and NAFLD. In a mouse model of high-fat diet-induced obesity, naringenin treatment suppressed obesity [143]. Naringenin lowered hepatic TG levels and increased the expression of hepatic fatty acid oxidation and ketogenesis regulators, such as PGC1α [143]. In a mouse model of methionine–choline deficiency diet-induced NAFLD, naringenin suppressed hepatic lipid accumulation and inflammation by inhibiting NLRP3/NF-κB pathway activation [144]. In high-fat diet-induced NAFLD mice, treatment with naringenin activated AMPK inhibited autophagy and lipid accumulation and increased energy expenditure [145]. Furthermore, in high-fat diet-induced NAFLD mice, naringenin suppressed weight gain and reduced TG and total cholesterol levels in the liver and blood [146].

**Atherosclerosis.** Atherosclerosis is characterized by plaque accumulation in the lining of the arteries and; in severe cases, it can lead to stroke and myocardial infarction [147, 148]. Cholesterol mainly accumulates in the form of LDL [149]. If accumulation continues, blood flow decreases, and hypoxia occurs [149].

By improving blood lipid levels and suppressing plaque accumulation, naringenin suppresses atherosclerosis. Naringenin reduced aortic plaque deposits in a Western diet mouse model [150]. Additionally, naringenin decreased TG and total cholesterol levels [150]. Ldlr^−/−^ mice fed a high-fat-cholesterol diet developed atherosclerosis, and naringenin treatment decreased plaque macrophages and increased smooth muscle cells [151]. By reducing plasma TG and cholesterol levels, naringenin inhibited plaque formation [151]. In ApoE^−/−^ mice with atherosclerosis and vascular aging, naringenin suppressed the excessive production of ROS and increased the activity of antioxidant enzymes in the aorta [152]. Furthermore, the increased SIRT1 activity by naringenin increased the deacetylation and protein expression of the downstream factors, FOXO3a and PGC1α [152]. The administration of naringenin (FA-LNPs/Nrg), an oral nanomedicine made through FA-LNPs encapsulation, to ApoE^−/−^ mice reduced the aortic lesion area and plaque areas [153]. Moreover, FA-LNPs/Nrg treatment reduced blood TG, total cholesterol, and LDL levels and increased HDL levels [153].

### Neurodegenerative Diseases

**Alzheimer’s disease.** AD refers to chronic and persistent memory loss that causes cognitive impairment [154]. AD is characterized by the formation of amyloid plaques and neurofibrillary tangles composed of amyloid-beta (Aβ) and hyperphosphorylated tau [154, 155]. Acetylcholinesterase (AChE), butyrylcholinesterase (BChE), and amyloid precursor protein cleaving enzyme 1 (BACE1) are essential enzymes responsible for AD development [156].

By regulating the PI3K/Akt/GSK-3β pathway and inhibiting AChE activity, naringenin suppresses memory loss. First, in AD-induced *in vivo* and *in vitro* models, naringenin activated the PI3K/Akt pathway and phosphorylated GSK-3β. In an AD rat model, naringenin treatment at 25, 50, and 100 mg/kg improved spatial learning and memory by regulating the PI3K/Akt/GSK-3β pathway and inhibiting tau hyperphosphorylation [157]. Additionally, naringenin increased PPAR-λ and insulin transport to the brain [157]. In PC12 cells, naringenin treatment 0.4 μM inhibited Aβ-induced AD apoptosis and neurotoxicity by PI3K/Akt/GSK-3β pathway activation and caspase-3 inhibition [158]. Second, in AD-induced *in vivo* and *in vitro* models, naringenin dose-dependently decreased AChE activity [159]. In an ICR mouse model of scopolamine-induced amnesia, the effects of naringenin 4.5 mg/kg treatment on amnesia were confirmed using the maze test and passive avoidance experiment [159]. In an *in vitro* study, naringenin lowered the activities of AChE, BChE, and BACE1 [160]. Furthermore, in an Aβ-induced AD mouse model, naringenin oral administration improved memory [161]. Moreover, naringenin inhibited apoptosis and lipid peroxidation by reducing the MDA level in the hippocampus [161].

**Parkinson’s disease.** PD is characterized by the loss of dopaminergic neurons in the midbrain [162]. PD causes motor disorders and may initially cause non-motor disorders, including anosmia, depression, and sleep disorders [163]. The regulation of neuroinflammation, dopamine, and oxidative stress play significant roles in PD [164]. 6-Hydroxydopamine (6-OHDA) is one of the neurotoxins used for inducing PD models by causing damage to dopamine neurons in the nigrostriatum [165]. Paraquat (PQ), a frequently used pesticide, induces oxidative stress and causes PD-like lesions in rodent animal models [166]. Rotenone-induced PD models can reproduce the main pathological features of clinical PD models [167].

Naringenin protects against oxidative damage caused by the PD inducers, including 6-OHDA, PQ, and rotenone. First, in 6-OHDA–induced *in vivo* and *in vitro* PD models, naringenin inhibited neurotoxicity by activating Nrf2/ARE signaling in SH-SY5Y cells [168]. Nigrostriatal dopaminergic neurodegeneration and oxidative damage caused by 6-OHDA were suppressed in mice administered with oral naringenin [168]. The 6-OHDA–induced PD rat model showed a loss of tyrosine hydroxylase (TH)-positive cells in the substantia nigra and decreased dopamine levels in the striatum; however, naringenin protected them [169]. Second, naringenin inhibited oxidative stress in PQ-induced *in vivo* and *in vitro* PD models. In SH-SY5Y cells with PQ-induced PD, naringenin treatment reduced oxidative damage and increased cell viability and ATP levels [170]. In a PQ-induced PD rat model, naringenin treatment suppressed behavioral disorders, mitochondrial dysfunction, and oxidative stress [170]. Moreover, naringenin increased the expression of TH, which is involved in dopamine synthesis [170]. In a PQ-induced PD rat model, PLGA nanoparticles loaded with naringenin increased the levels of SOD, GSH, CAT, and brain-derived neurotrophic factor and decreased the levels of MDA and α-Synuclein protein [171]. Third, in a rotenone-induced *in vivo* PD model, naringenin increased the activity of antioxidant enzymes and improved motor dysfunction. In a rotenone-induced PD model, naringenin 50 mg/kg pretreatment restored motor and non-motor impairment, thereby increasing the activity of antioxidant enzymes [172]. Furthermore, in a rotenone-induced PD rat model, naringenin 10 mg/kg treatment protected the neuronal morphology and damage [173]. In addition, naringenin restored motor capacity and body weight and increased the levels of parkin, C terminus Hsp70 interacting protein, PARK 7 protein (DJ1), and TH in the substantia nigra and striatum [173]. In summary, naringenin inhibited oxidative damage and other factors caused by 6-OHDA, PQ, and rotenone, which are representative inducers of PD.

## Conclusion and Future Prospects

Naringenin is a flavanone and is a well-known polyphenol. Naringenin has various physiological activities and has been shown to positively affect colorectal, gastric, lung, breast, ovarian, cervical, prostate, bladder, liver, pancreatic, and skin cancers. Moreover, naringenin has been shown to positively influence metabolic disorders, including diabetes, obesity, hyperlipidemia, hypertension, hyperglycemia, steatosis, liver disease, and atherosclerosis, as well as neurodegenerative diseases, including AD and PD.

According to clinicaltrials.gov, clinical studies on naringenin and citrus fruit extracts are actively underway. First, clinical studies using extracts have confirmed the safety and pharmacokinetics of naringenin. The serum naringenin concentration was confirmed after the oral administration of citrus extracts. Second, a clinical study using naringenin has been conducted. A study on whether naringenin can prevent hepatitis C virus infection and the effects of naringenin administration on subjective cognitive decline is ongoing. Furthermore, a study has investigated the effects of naringenin combined with β-carotene on energy consumption and glucose metabolism. In this manner, clinical trials on naringenin are actively underway.

These studies have suggested that naringenin can be implemented in clinical trials for the diseases introduced in this review. Overall, naringenin may become a promising preventive and therapeutic option for diseases threatening human health.
